# Synthesis, Conformational Analysis and Antitumor Activity of the Naturally Occurring Antimicrobial Medium-Length Peptaibol Pentadecaibin and Spin-Labeled Analogs Thereof

**DOI:** 10.3390/ijms241713396

**Published:** 2023-08-29

**Authors:** Laura Morbiato, Celeste Quaggia, Luca Menilli, Chiara Dalla Torre, Antonio Barbon, Marta De Zotti

**Affiliations:** 1 Department of Chemical Sciences, University of Padova, 35131 Padova, Italy; laura.morbiato.1@unipd.it (L.M.); celeste.quaggia@studenti.unipd.it (C.Q.); chiara.dallatorre.2@studenti.unipd.it (C.D.T.); antonio.barbon@unipd.it (A.B.); 2 Department of Biology, University of Padova, 35131 Padova, Italy; luca.menilli@unipd.it

**Keywords:** antitumor peptide, breast cancer, α-helical conformation, peptaibol, peptide–membrane interaction

## Abstract

Peptaibols are proteolysis-resistant, membrane-active peptides. Their remarkably stable helical 3D-structures are key for their bioactivity. They can insert themselves into the lipid bilayer as barrel staves, or lay on its surface like carpets, depending on both their length and the thickness of the lipid bilayer. Medium-length peptaibols are of particular interest for studying the peptide–membrane interaction because their length allows them to adopt either orientation as a function of the membrane thickness, which, in turn, might even result in an enhanced selectivity. Electron paramagnetic resonance (EPR) is the election technique used to this aim, but it requires the synthesis of spin-labeled medium-length peptaibols, which, in turn, is hampered by the poor reactivity of the C^α^-tetrasubstituted residues featured in their sequences. After several years of trial and error, we are now able to give state-of-the-art advice for a successful synthesis of nitroxide-containing peptaibols, avoiding deleted sequences, side reactions and difficult purification steps. Herein, we describe our strategy and itsapplication to the synthesis of spin-labeled analogs of the recently discovered, natural, medium-length peptaibol pentadecaibin. We studied the antitumor activity of pentadecaibin and its analogs, finding potent cytotoxicity against human triple-negative breast cancer and ovarian cancer. Finally, our analysis of the peptide conformational preferences and membrane interaction proved that pentadecaibinspin-labeling does not alter the biological features of the native sequence and is suitable for further EPR studies. The nitroxide-containing pentadecaibins, and their synthetic strategy described herein, will help to shed light on the mechanism of the peptide–membrane interaction of medium-length peptaibols.

## 1. Introduction

**Figure 1:**
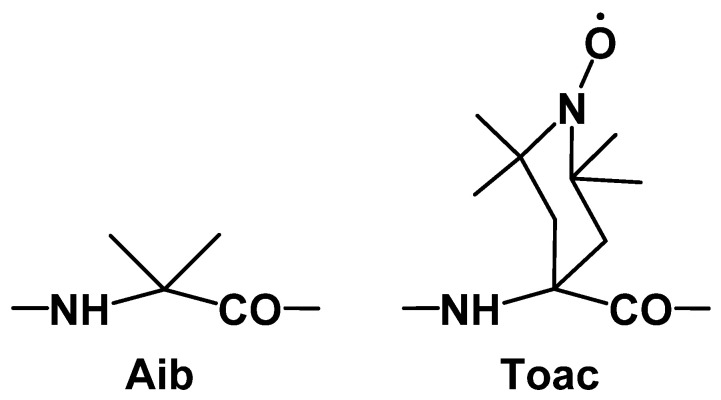
Chemical structures of C^α^-tetrasubstituted α-amino acid residues Aib and Toac.

The WHO states that “Cancer is a leading cause of death worldwide, accounting for nearly 10 million deaths in 2020” [1,2]. A total investment in cancer research of about USD 24.5 billion was received in the timeframe of 2016–2020 [3]. In this framework, antimicrobial peptides (AMPs) [4] are very promising active agents [5,6,7], since they usually act on the cell membrane and are less likely to induce drug resistance in cancer cell lines [8]. Indeed, peptides can be used to overcome limitations of current anticancer therapies [9,10]. On the other hand, AMPs suffer from a lack of proteolytic stability and poor selectivity between healthy cells and cancer cells [11]. Peptaibols [12,13] are naturally occurring, helical AMPs with a strong resistance to proteolysis [14,15]. Their sequences are characterized by the presence of many C^α^-tetrasubstituted Aib (α-aminoisobutyric acid; Figure 1) residues, a C-terminal 1,2-aminoalcohol and an N-terminal acyl group. They possess antimicrobial [16,17], antiviral [18] and antitumor [19,20] activity, even against drug-resistant cancer cell lines [21,22] and bacteria [23]. They are known to exert their bioactivity by interacting with the phospholipid membranes [24,25,26] through different mechanisms, depending mainly on the match between the thickness of the target membrane and the length of their helical 3D structure, which, in turn, depends on their sequence length.

The membrane-disrupting properties of peptaibols represent an attractive feature to target hyperproliferative diseases such as cancer. Additionally, peptaibols bioactivity does not rely on the interaction with proteins such as ATP-binding cassette (ABC) transporters, which are known to develop multi-drug resistance, and peptaibols have already shown potential for application in chemotherapy-resistant tumors [21,22].

Peptaibols are qualified as short-, medium- or long-length, depending on the number of residues in their sequence. Short-length peptaibols (5–11 residues) are commonly believed to act through a carpet mechanism [27], while long-length peptaibols (18–20 residues), such as the well-known alamethicin, act through the barrel stave mechanism, in which a number of peptide molecules form a helical bundle in the membrane, often generating voltage-dependent pores [28,29]. Medium-length peptaibols (14–16 residues) are peculiar, since their length ideally allows them to switch between the two mechanisms depending on the membrane thickness and composition, suggesting that they might display improved selectivity compared with other membrane-active peptides. Therefore, they are ideal for shedding light on the mechanisms of the peptide–membrane interaction, with the final aim of improving peptide selectivity and cytotoxicity against cancer cell lines. To this aim, electron paramagnetic resonance (EPR) is one of the most informative spectroscopic techniques commonly employed to study peptide orientation and self-assembly in phospholipid membranes [30,31,32]. To exploit EPR, peptides must be spin labeled, e.g., by inserting a nitroxide-containing moiety. Clearly, in order to obtain reliable data, such spin labeling must not alter the bioactivity and membrane interaction mechanism of the native peptide [33] or be too flexible [34,35]. The best spin label for studying peptaibols via EPR is the nitroxide-containing C^α^-tetrasubstituted α-amino acid Toac (2,2,6,6-tetramethylpiperidine-1-oxyl-4-amino-4-carboxylic acid; Figure 1) [36]. It can be inserted in the peptaibol sequence instead of an Aib, since they share the same conformational preference for helical structures [37,38,39,40].

Despite their promising characteristics, medium-length peptaibols are not widely exploited by the scientific community, essentially because their synthesis is complicated by two factors: (i) the poor reactivity of Aib is worsened by the presence of several Aib residues in a row [40], and (ii) they usually contain the dipeptide motif Aib-Pro (or Hyp) [41,42] that is both acid-labile [42] and prone to form a diketopiperazine adduct during step-by-step synthesis, leading to the loss of the two residues from the growing peptide chain [43]. On the other hand, recently, the naturally occurring medium-length peptaibol pentadecaibin was discovered [44]. Its sequence does not contain Aib-Pro/Hyp motifs. Still, three Aib residues in a row are present at its C-terminus (Table 1). We recently reported the synthesis and 3D structure analysis of the native sequence pentadecaibin [45]. By building on the strategy reported there and on our past experience in the insertion of spin-labeled residues in peptides, we herein report the synthesis of two pentadecaibin analogs in which Aib residues at position 1 or 11 were replaced with Toac. Circular dichroism analysis in different environments and a fluorescent dye release assay from liposomes allowed us to confirm that such sequence modification does not alter the 3D structure and membrane interaction ability of the native pentadecaibin. Finally, we demonstrate the potential of medium-length peptaibols as anticancer agents by reporting on the cytotoxicity of pentadecaibin and its analogs against several cancer cell lines.

## 2. Results

### 2.1. Peptide Synthesis

The synthesis of spin-labeled medium-length peptaibols is complicated by a number of factors. Both Aib and Toac (Figure 1) are very poorly reactive amino acid residues, but especially the latter. When Toac has to act as a nucleophile onto an activated Aib, the reaction simply does not proceed. Indeed, all our attempts to synthesize a pentadecaibin analog containing Toac at position 13 via solid-phase peptide synthesis (SPPS) were totally unsuccessful, with no traces of the desired product regardless of the activation conditions or reaction times. On the other hand, the quantitative insertion of Toac onto Aib was achieved via a 24 h reaction with three equivalents of Fmoc-Toac-F (Fmoc, fluorenylmethoxycarbonyl) obtained via a 15 min preactivation of Fmoc-Toac-OH with tetramethylfluoroformamidinium hexafluorophosphate (TFFH) and diisopropylethylamine (DIPEA).

Toac is also sensitive to acidic conditions. Acidic treatments result in disproportionation with radical protonation that can be reversed by a reaction with aq. NH_4_OH 33%, but this additional step requires further purification, leading to product loss. Additionally, when the acidic treatment is strong (e.g., >90% trifluoroacetic acid, TFA) the quantitative recovery of the free radical moiety is usually not achieved, again reducing the yield.

Moreover, in the synthesis of Toac-containing pentadecaibins (Table 1) and, previously, heptaibins [46], we faced issues connected with the presence of Gln and Hyp residues, respectively, but the same holds true for Ser residues, e.g., in tylopeptin [39]. At first, we performed the synthesis with protected side chains using trityl (Trt) and benzyl protecting groups for Gln or Hyp, respectively. In both cases, this was not a good choice, since the removal of side-chain protections led to difficult-to-purify crude mixtures.In particular, the deprotection of the Gln side chain inevitably generates by-products.

Treatment with 1,1,1,3,3,3-hexafluoroisopropanol (HFIP) 30% in CH_2_Cl_2_ cleaves the peptide from the 2-ClTrt resin (preloaded with Phol) but does not remove the Trt group from the Gln side chain. Its use resulted in a crude product of high purity (Figure S1, Supplementary Materials). Many side-products were conversely detected during subsequent side-chain protection removal, likely originating from acid-induced peptide degradation, to the point that in some cases, the desired peptide is no longer detectable in the resulting mixture. We particularly noted the presence of two by-products, with masses corresponding to [M-18] and [M-58], respectively, which were reproducibly detectable during side-chain protection removal. [M-18] may arise from the dehydration of one Gln side chain amide group to nitrile, a side-reaction commonly found for Asn residues, but not reported for Gln. The second mass is not easily interpreted; it seems to imply amide loss from Gln residues, but a plausible mechanism could not be devised. We performed many attempts to achieve peptide cleavage from the resin and side-chain deprotection (a couple of examples of mass spectra of the resulted complex mixtures are reported in Figures S2 and S3, Supplementary Materials). Any treatments aiming to obtain concurrent peptide cleavage from the resin and side-chain deprotection led to very complex crude mixtures.

Finally, we found that the best procedure in terms of crude purity and yield consists of two steps: (i) peptide cleavage from the chlorotrityl resin with HFIP 30% in CH_2_Cl_2_ for 1 h (repeated treatments led to a quite modest increase in the yield, so they are not very useful) and (ii) treatment of the dry crude peptide obtained with a cocktail of TFA 5% and triisopropylsilane (TIS) 2.5% in CH_2_Cl_2_ in a round-bottomed flask for 7 hours (less time resulted in incomplete Trt removal, more time led to byproduct formation, and the addition of water to the cocktail led to more byproducts, so it should be avoided; Table 2). The high-resolution mass spectrometry (HRMS) spectrum of the crude peptide obtained using this method is reported in Figure S4, Supplementary Materials.

Although the described method allowed us to obtain fairly good crude mixtures, the crude peptaibols are not easily purified from free Trt that remains in the mixture even after repeated diethyl ether precipitation steps. This difficulty is mainly due to peptide hydrophobicity, which makes their chromatographic behavior very similar (in other words, peptide and Trt usually elute together). Finally, when TFA is used, special care must be taken to avoid the esterification of the C-terminal Phol of peptaibols. We found that this side reaction can be completely avoided by pouring the cleavage mixture from the reaction vessel directly onto a vial containing 1–2 mL of CH_3_OH.

Even after applying all the tricks, the presence of Trt-adducts combined with the acid-induced Toac protonation needing regeneration and the by-product formation mentioned above led to complex crude mixtures and a very poor final yield (about 2% after purification). Clearly, this cannot be considered a good strategy. Therefore, we performed the synthesis again by trying different routes, in particular, three alternative combinations of active agents (Oxyma-Pure/diisopropylcarbodiimide (DIC), 1H-1,2,3-triazole [4,5-b]pyridinium 3-oxid hexafluorophosphate (HATU)/1-hydroxy-7-azabenzotriazole (HOAt)/DIPEA or TFFH/DIPEA); three reaction times (2 h, overnight, or 24 h); double/triple coupling steps; and protected/unprotected amino acid side chains. We found that the best procedure in terms of yield and crude purity includes a 24 h reaction time and TFFH activation for Aib/Toac onto Aib couplings, and the use of Gln residues unprotected at the side chain. Glutamine can be used without side-chain protection, but there is the well-known risk of its dehydration to nitrile when carbodiimides are used as coupling reagents [47,48]. However, by using unprotected Gln, not only we did not detect any byproduct during the synthesis, but we could also speed up the whole procedure, since with a 1 h treatment with 30% HFIP in CH_2_Cl_2_, we obtained a product with good yield (ca. 60%) and crude purity (>75%) without affecting the Toac nitroxide radical moiety (HRMS spectrum and HPLC chromatogram of the crude product are reported in Figure S5, Supplementary Materials). The crude product was then easily purified to >93% via a simple step in medium-pressure liquid chromatography (Isolera Prime system).

**Scheme 1:**
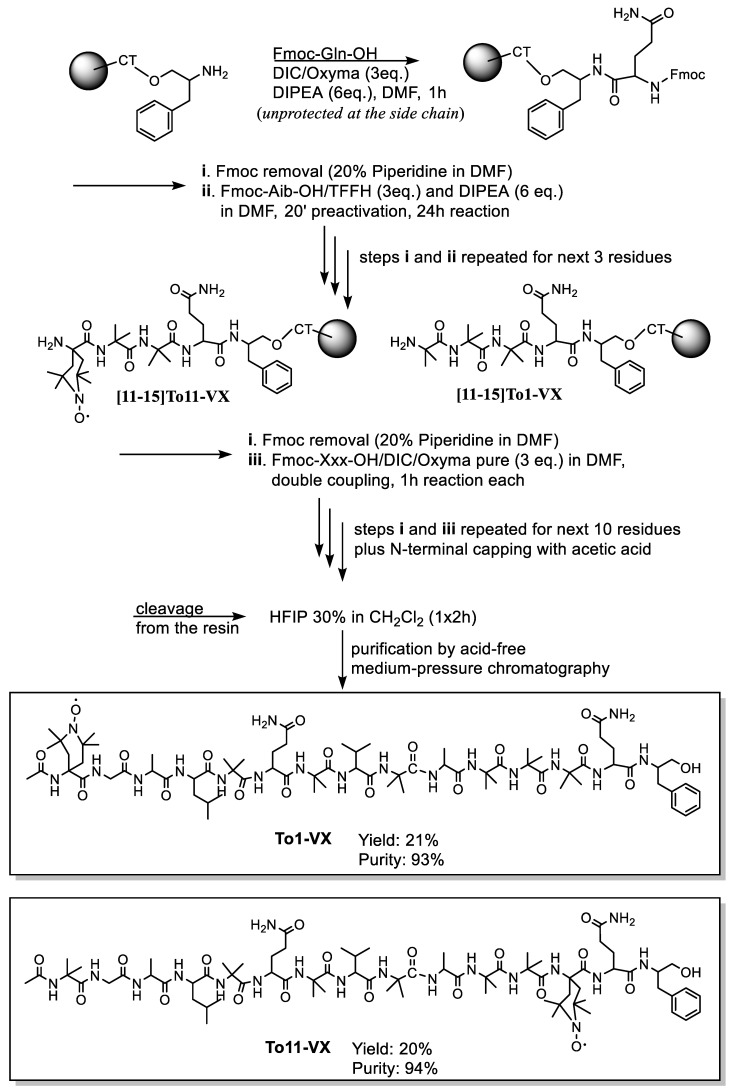
Protocol for the chemical synthesis of To1-VX and To11-VX.

Thanks to this procedure, we obtained the two Toac-containing pentadecaibin analogs To1-VX and To11-VX (Table 1) with a final yield of about 20%, after purification to >93% (HRMS spectra and HPLC profiles of the purified products are reported in Figures S7–S10, Supplementary Materials). Our best synthetic procedure is reported in Scheme 1.

Our considerations on the synthesis of Toac-containing pentadecaibins can be extended to other peptaibol sequences and are summarized in Table 2.

### 2.2. Electron Paramagnetic Resonance Characterization

A continuous-wave EPR analysis confirmed the presence of the radical moiety after purification for the two monolabeled peptides To1-VX and To11-VX (Figure S6, Supplementary Materials). The measured electronic gyromagnetic factor (g ± 0.0001) for the compounds are To1 − VX g = 2.0067 and To11 − VX g = 2.0063.

### 2.3. Circular Dichroism (CD)

**Figure 2:**
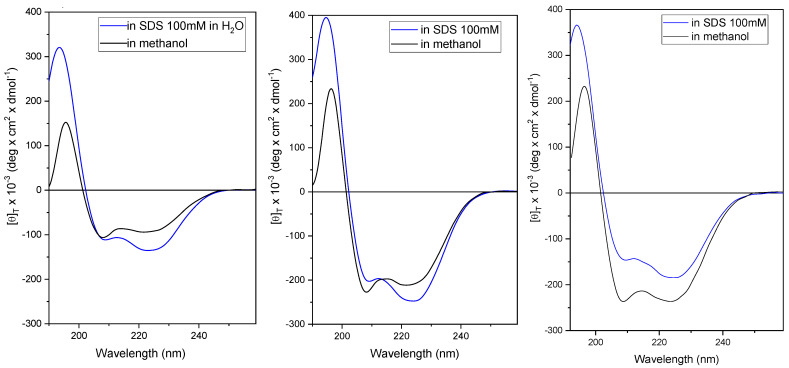
CD spectra of pentadecaibin (left), To1-VX (middle) and To11-VX (right). All peptides are dissolved in either 100 mM aqueous SDS (blue) or CH_3_OH (black) at 10^−4^ M concentration.

We performed a CD analysis of the peptides in methanol and in the presence of micelles of sodium dodecyl sulfate (SDS) 100 mM in water. Those conditions allowed us to carefully investigate the conformational preferences of the peptaibols (i) as monomers in a solvent that does not support any particular 3D structure (methanol) and (ii) in a membrane-mimicking environment (aqueous SDS 100 mM), where the peptides should adopt their bioactive structure, perhaps aggregating in helical bundles (self-assembly induced by peptide–membrane interaction). Figure 2 shows the CD spectra of pentadecaibin and its analogs in the two different solvents.

CD spectra of α-helical peptides feature two negative and one positive maxima centered at about 208, 222 and 195 nm, respectively [49]. Aib-containing peptides are known to fold in 3_10_-helices, which is common for peptaibols. In this case, the first negative maximum shifts from 208 to about 205 nm [50]. Additionally, the intensities of the two negative maxima are similar for α-helices, while the band at 205 is significantly more intense that that at 222 nm for a 3_10_-helix [50].

From the analysis of the dichroic profiles obtained for the pentadecaibins (Figure 1), a difference can be observed between the relative intensities of the two negative maxima (208 nm and 222 nm) as the used solvent varies. The presence of a predominantly right-handed α-helical structure in both CH_3_OH and SDS is demonstrated by the position and sign of the bands. Indeed, all CD spectra show three maxima, including two negative maxima centered at approximately 208 nm and 222 nm and one positive maximum centered at about 195 nm. The ratio of molar ellipticities in CH_3_OH *R* = [θ]_222_/[θ]_208_ ≈ 0.9 is typical of the α-helix. In the membrane-mimetic environment (SDS), the ratio *R* = [θ]_222_/[θ]_208_ becomes greater than 1, which is an index of the presence of aggregation between helices, likely induced by the presence of the micelles. As for the dichroic profiles of the peptide analogs To1-VX and To11-VX, they show the same trend as the native sequence. Both in methanol and in SDS, the positions of the dichroic bands are diagnostic of the presence of a right-handed α-helical structure, with a ratio *R* between the molar ellipticities in CH_3_OH [θ]_222_/[θ_]208_ ≈ 0.9. In the membrane-mimetic environment (SDS), the [θ]_222_/[θ]_208_ ratio becomes higher than 1, again indicating the presence of aggregation in this case. In conclusion, the synthesized analogs assume stable right-handed α-helical structures, close to that of the native sequence pentadecaibin. This behavior is mainly due to the known common ability to induce helical structures of the two C^α^-tetrasubstituted residues, whereby the substitution of the Aib residue with Toac does not alter the native peptide conformation.

**Figure 3:**
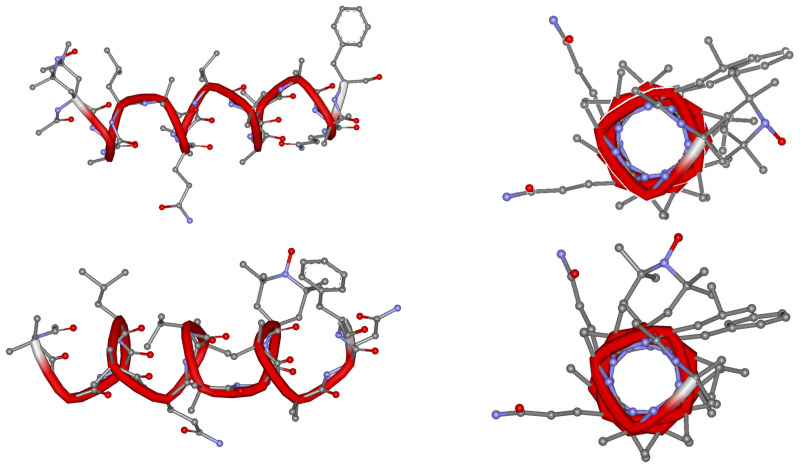
Three-dimensional models of To1-VX (**top**) and To11-VX (**bottom**) in α-helical conformation (red ribbon), built with the software HyperChem 6 and visualized with WebLab ViewerPro 3.7, with views along and perpendicular to the helical axes. The oxygen and nitrogen atoms are shown in red and blue, respectively.

We built 3D models of the two peptides in an α-helical conformation (Figure 3). The α-helix is clearly amphipathic, with the two Gln residues lying on the same less-hindered face. Toac is at the hydrophobic side of both analogs so as to minimize the perturbation of the polarity balance of the tridimensional structure that is crucial for peptide–membrane interaction.

### 2.4. Peptide-Induced Leakage from Model Liposomes

The leakage assay allowed us to verify the ability of peptides to modulate the membrane permeability of model liposomes, giving an indication of the efficacy of the peptide–membrane interaction, which is crucial in the mechanism of the biological action of peptaibols.

The assay was carried out on model liposomes (small unilamellar vesicles, SUV) of two different compositions, namely phosphatidylcholine/cholesterol (PC/Ch) 7:3 and phosphatidylethanolamine/phosphatidylglycerol (PE/PG) 7:3. Using fluorescence measurements, the ability of the peptides to induce the release of the carboxyfluorescein (CF) dye previously included within SUVs was evaluated.

**Figure 4:**
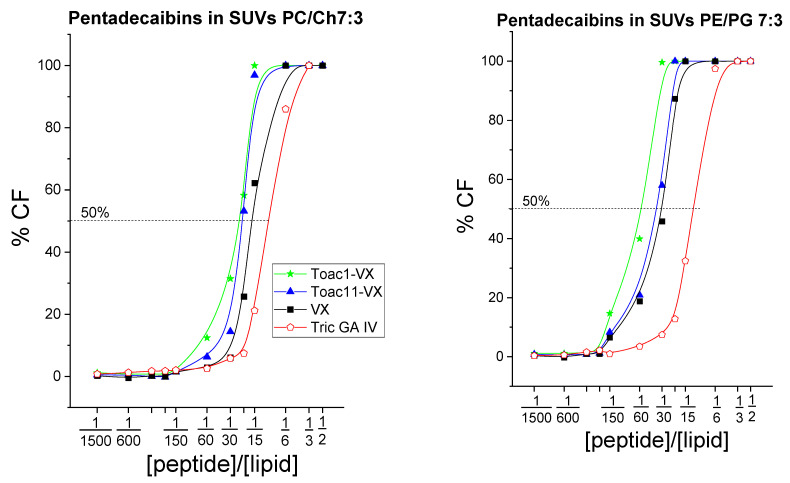
Results of the leakage assay performed on pentadecaibin and its Toac-containing analogs at increasing peptide-to-lipid ratio. SUV composition: left PC/Ch 7:3; right PE/PG 7:3. %CF, release (% over total release caused by Triton) of encapsulated CF dye from SUVs.

The chosen lipid compositions result in different net surface charges on the SUVs. In particular, PC/Ch liposomes are zwitterionic and contain cholesterol, mimicking eukaryotic cells, while in those of the PE/PG type, a net negative charge is created on the surface thanks to the presence of PG, as in cancer cells. Being tests on model liposomes, the results of these experiments are to be considered entirely qualitative. If the peptide does not interact with the membranes, the CF is not released, and therefore, no fluorescence emission due to the removal of the self-quenching phenomenon is observed (in turn, due to the high concentration of CF inside the liposomes). On the other hand, when the peptide perturbs the artificial membrane, it causes the leakage of the CF, and therefore leads to an increase in fluorescence intensity. The maximum value of fluorescence intensity, corresponding to the complete destruction of the vesicles via the surfactant Triton X-100, was used as a reference. Figure 4 shows the results of the tests that were carried out. The short-length antitumor peptaibol trichogin GA IV (Tric GA IV) [21] is reported as a positive control, and the graphs are shown in logarithmic scale to highlight the sigmoid trend of the curves.

The results reported in Figure 2 demonstrate that pentadecaibin (VX) and its Toac-containing analogs are all equally membrane active, with a better efficiency in causing membrane leakage than trichogin, particularly on the negatively charged PE/PG SUVs. This is not surprising, since pentadecaibin has a longer helical structure than trichogin, and can modify its membrane interaction mechanism in response to differences in the target lipid membrane. Clearly, the ability of the peptides to cause leakage from model liposomes cannot provide an indication of the mechanism of peptide–membrane interaction. The EPR of the newly synthesized Toac-containing peptaibols will hopefully help shed light on the mechanism of pore formation.

### 2.5. In Vitro Activity of Pentadecaibins against Human Cancer Cells

**Figure 5:**
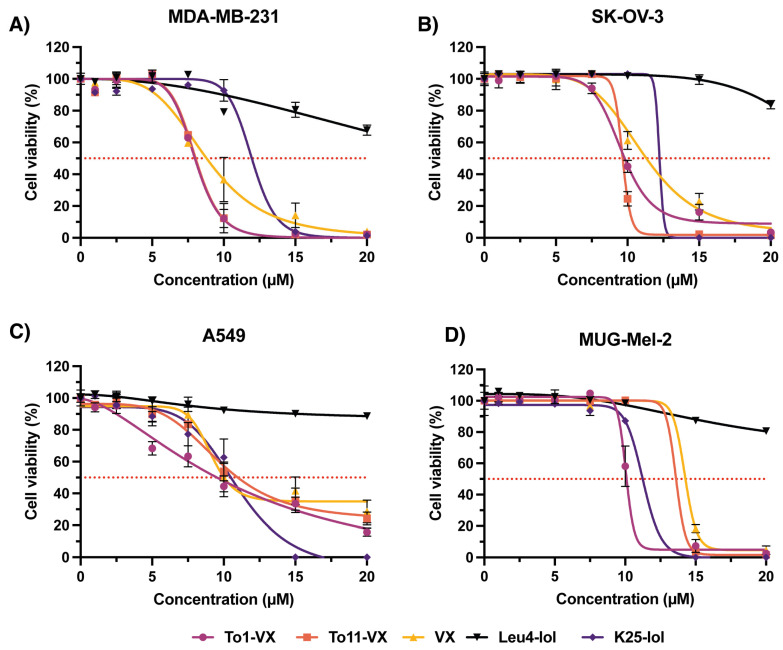
Dose–response curves of MDA-MB-231 (**A**), SK-OV-3 (**B**), A549 (**C**) and MUG-Mel2 (**D**). Cells were exposed for 24 h to increasing concentrations of pentadecaibin and its analogs, as well as two known peptaibols as positive (K25-lol) and negative (Leu4-lol) controls. Cell viability was measured using the MTS at the end of the incubation time. Data are expressed as mean percentage ± SD of at least three independent experiments, carried out in triplicate.

The in vitro anticancer activity of VX and its analogs To1-VX and To11-VX was assessed using the MTS assay in MDA-MB-231 (human triple-negative breast cancer), A549 (human lung adenocarcinoma), SK-OV-3 (human ovarian adenocarcinoma) and MUG-Mel2 (human melanoma) cancer cell lines. Two peptaibols, known to be non-cytotoxic (Leu-lol) and antitumor (K25-lol) [22], were included as negative and positive controls, respectively. Dose–response curves were generated (Figure 5), and the half maximal inhibitory concentration (IC_50_) values were calculated for each peptaibol (Table 3).

For all peptaibols, a concentration-dependent reduction in cell viability was observed. The antiproliferative activity of the To1-VX and To11-VX peptaibols was comparable to VX and to the positive control K25-lol for all the tested cell lines. At the highest concentration tested, all the peptaibols induced a 100% cell viability reduction in all cancer cell lines except for the A549 cell line, where the activity of all three peptaibols reached a maximum of 70–80% reduction in cell viability. MDA-MB-231 was the most sensitive cell line to the three peptaibols of all those tested, with an IC_50_ of about 8 µM for To1-VX and To11-VX and 9 µM for VX. Also, in the ovarian carcinoma line SK-OV-3, it was possible to observe a notable antiproliferative activity induced by the peptaibols. The MUG-Mel2 melanoma cell line was the least sensitive to the three peptides of those tested.

## 3. Discussion

This work focused on pentadecaibin, a medium-length peptaibol. The synthesis strategy was optimized to obtain spin-labeled pentadecaibin analogs, containing the nitroxide-bearing Toac residue, which replaces Aib^1^ or Aib^11^. The main goal was to reduce byproduct formation and to shorten the production and purification procedures compared to previous strategies [39,45].

The use of the TFFH activator for the insertion of Aib or Toac onto Aib allowed for the optimal synthesis of the sequence, providing a significantly better result than the standard DIC and Oxyma-Pure activators [51]. The exploitation of Gln with unprotected side chains resulted in an increase in both the crude purity and yield.

^1^H 1D NMR characterization was initially not performed on the peptides because radicals (such as the nitroxide group on Toac) are known to suppress NMR signals. On the other hand, in the two analogs studied herein, Toac is at, or near, a peptide terminus. Thus, a signal could be obtained for the protons that were not close to the spin label, which are clearly more for To1-VX than To11-VX (Supplementary Materials, Figures S11 and S12). It is interesting to note the radical-induced absence of the acetyl signal at about 2 ppm for To1-VX, which was instead detectable for To11-VX.

The synthesized peptides adopt a right-handed α-helix, which is in line with what was reported for long- and medium-length peptaibols [37,39,40], with a clear, albeit modest, amphiphilic character, again a common feature, believed to improve peptide affinity to phospholipid membranes [31]. In the presence of SDS micelles, the α-helices aggregate, as indeed expected from their known ability to interact with the membranes of the peptides belonging to the peptaibol family.

The sigmoid shape of the curves of CF release from the SUVs reported in Figure 4 demonstrates the onset of a cooperative mechanism, whereby it is necessary to reach a certain threshold concentration for the peptides to exhibit membrane-interacting activity. This aspect is typical of peptaibols and is linked to the formation of helical aggregates in the presence of membranes, also evidenced by the abovementioned CD studies in the presence of SDS micelles. In conclusion, the assay confirms the ability of pentadecaibin and its analogs to interact with membranes, modifying their permeability. This ability likely contributes to their antitumor action.

Pentadecaibin and the analogs To1-VX and To11-VX showed promising antiproliferative activity, with IC_50_ in the low micromolar range against triple negative breast cancer (MDA-MB-231) and ovarian cancer (SK-OV- 3) cell lines. The high activity of the peptides against the in vitro model MDA-MB-231, compared to other peptides [52], is of note, since the diagnosis of triple-negative breast cancer, especially among young women, has dramatically increased [53,54]. Moreover, since this subtype does not possess important hormonal receptors, such as those for estrogen, progesterone and human epidermal growth factor receptor 2 (HER2) [55,56], hormonal therapies are almost ineffective against it. The development of new molecules with potent activity against this breast cancer subtype is thus of particular interest.

Together with a direct effect, the peptaibol-promoted disruption of membrane integrity can induce an important influx of ions into the intracellular environment [57]. For instance, Ca^2+^ ions play an important role in initiating apoptotic signaling. As a possible signaling pathway, a peptaibol-induced increase in intracellular Ca^2+^ may be enough to activate calpain, an intracellular cysteine protease that plays a major role in initiating Ca^2+^-triggered, caspase-dependent cell death. The activation of calpains may also result in the translocation of Bax to the mitochondria, ultimately leading to intrinsic apoptosis pathway activation and cleavage of Bid, resulting in the release of cytochrome c from the mitochondria [58].

Medium-length peptaibols have the potential to modify their mechanism of interaction with membranes depending on the thickness and lipid composition of the membranes themselves. The Toac-containing pentadecaibin analogs, produced with good yield (20%) and purity (>93%), will be exploited in further EPR measurements to shed light on the intriguing mechanism of the peptide–membrane interaction of this promising class of bioactive peptides.

## 4. Materials and Methods

### 4.1. Peptide Synthesis

Fluorenylmethyloxycarbonil-(Fmoc-)protected amino acids, TFFH active agent, DMF and HPLC solvents for peptide synthesis were Sigma-Aldrich products, used as received. Oxyma-pure and DIC were purchased from IRIS Biotech. All other reagents were obtained from Merck (Boston, MA, USA).

The synthesis of Toac-containing pentadecaibins foresees the use of unprotected side-chain Gln residues and the use of TFFH to couple Toac, as described in the text, and the procedure is similar to that reported in [45]. Briefly, the manual solid phase peptide synthesis was carried out on a Phol-preloaded 2-chlorotrytil resin (loading: 0.4 mmol/g). N,N-dimethylformamide (DMF) was used as solvent. When Aib was not involved, single coupling was performed with 3 equivalents of the Fmoc-amino acid residue, DIC and Oxyma Pure. Aib^1^ was acetylated via multiple treatments with acetic acid, Oxyma pure and DIC. After shrinking of the resin with anhydrous dichloromethane (DCM), peptide was cleaved via a 2 h treatment with a solution of HFIP 30% in DCM. This mild and acid-free treatment leaves Toac spin label unaffected. The crude peptides were purified via medium-pressure reverse-phase chromatography (Biotage, Uppsala, Sweden, Isolera Prime instrument) using acid-free eluants (A: water; B: acetonitrile/water 9:1) with the UV detector operating simultaneously at two wavelengths, 260 and 206 nm, and obtained with a purity ≥ 93%. The crude sample was loaded into a C_18_ Duo Sfär column (12 g) through the corresponding loading cartridge. The flow was set to 12 mL/min. Before loading the sample, the column was equilibrated by eluting 4 CV (column volume = 15 mL) of 100% B, 4 CV of 50% A and 50% B and 4 CV 100% A. Then, the sample was loaded into the cartridge using a maximum volume of 1 mL of CH_3_OH. A step gradient was used, with ramps interspersed with isocratic steps (Figure S13, Supplementary Materials). The following gradient was applied: 0%B, 6 CV; 0–30%B in 3 CV; 30%, 1 CV; 30–50%B in 2 CV; 50%B, 3 CV; 50–60%B in 2 CV; 60%B, 2 CV; 60–70%B in 2 CV; 70%B, 3 CV; 70–76%B in 2 CV; 75%B, 2.3 CV; 75–80%B in 1.9 CV; 805 B, 1 CV; 80–85%B in 0.4 CV; 85%B, 0.7 CV; 85–90% in 0.5 CV; 90%B, 1 CV; 90–97%B in 1.3 CV; 97–100%B in 0.6 CV; 100%B, 4 CV. A representative report of the purification procedure applied for To1-VX is reported in Figure S13, Supplementary Materials. HPLC (Agilent 1260, Santa Clara, CA, USA) chromatograms, electron spray ionization high-resolution mass spectrometry (ESI-HRMS) (Waters Micromass Xevo instrument, Milford, MA) spectra and ^1^H NMR (nuclear magnetic resospectra) for the synthesized peptides are reported in the Supplementary Materials. ^1^H 1D NMR was performed in CD_3_OH at 298 K on a Bruker AVANCE DRX-400 instrument (400 MHz for 1H). Peptide concentration: 0.8 mM.

To1-VX. HPLC (Phenomenex C_18_ column (Jupiter, 250 × 4.6 mm 5μm); 5–95%B in 40 min); retention time, R_t_: 39.28 min; ESI-HRMS: MW_calc_ 1567,9212; [M + H]^+^_found_ = 1568,9043; ^1^H NMR (400 MHz, 298 K, CD_3_OH) δ, ppm: 8.56, 8.28, 8.21, 8.12, 8.08, 7.93, 7.81, 7.61, 7.58, 7.44, 7.36, 7.34, 7.26, 7.24, 7.22, 7.18, 7.16, 6.76, 6.67, 4.17, 4.15, 4.08, 4.06, 3.93, 3.67, 3.50, 3.15, 3.00, 2.97, 2.95, 2.93, 2.92, 2.87, 2.83, 2.80, 2.77, 2.50, 2.42, 2.40, 2.38, 2.36, 2.25, 2.21, 2.15, 2.10, 2.10, 2.08, 1.90, 1.68, 1.63, 1.61, 1.59, 1.56, 1.54, 1.53, 1.52, 1.30, 1.09, 0.97.

To11-VX. HPLC (Phenomenex C_18_ column (Jupiter, 250 × 4.6 mm 5 μm); 5–95%B in 60 min) R_t_: 54.52 min; ESI-HRMS: MW_calc_ 1567.9212; [M + H]^+^_found_ = 1568.8862; ^1^H NMR (400 MHz, 298 K, CD_3_OH) δ, ppm: 8.79, 8.71, 8.32, 8.00, 7.84, 7.52, 7.43, 7.25, 6.76, 4.24, 3.88, 3.84, 3.75, 3.71, 3.33, 3.00, 2.87, 2.49, 2.23, 2.16, 2.05, 1.95, 1.91, 1.62, 1.59, 1.53, 1.51, 1.49, 1.30, 0.93.

### 4.2. EPR

The continuous-wave electron paramagnetic resonance (cw-EPR) spectra were obtained using a Bruker Elexsys spectrometer equipped with a MD5 dielectric cavity connected to a Flexline support. Microwave power was 20 mW to avoid saturation, and modulation frequency was 100 kHz and modulation amplitude was 1.0 G to avoid lineshape distortion. Acquisition time for a single spectrum was 20 s, while the number of scans acquired was 3 to improve signal-to-noise ratio. The measurement of the g-factor was conducted via comparison with a 7,7,8,8-tetracyanoquinodimethane lithium salt (LiTCNQ) powder sample, for which the g-factor is known with high precision [59].

### 4.3. CD Measurements

CD measurements were acquired on a Jasco J-1500 spectrophotometer (Tokyo, Japan), using a 1 mm pathlength quartz cell (Hellma Analytics, Munich, Germany). Methanol or a solution of sodium dodecyl sulfate (SDS) 100 mM in milliQ water was used as solvent. An amount of 1 mM of solutions of the three peptides in each of the two solvents of choice was prepared. Then, 300 µL of each solution was inserted in the 1 mm quartz cell and the cell put into the cell holder of the Jasco J-1500 instrument. The measurements were performed at 25 °C, acquiring 16 scans. The temperature was kept constant by means of an external Peltier thermostat (ThermoHaake C25P, Karlbruhe, Germany).

### 4.4. Leakage Assay

Phosphatidylcholine (PC), phosphatidylglycerol (PG), phosphatidylethanolamine (PE) and cholesterol (Ch) were purchased from Avanti Polar Lipids (Alabaster, AL, USA). Two lipid films were prepared, combining PC with Ch, and PE with PG at a 7:3 *w*/*w* ratio, respectively. The lipids, dissolved in chloroform in a test tube and dried over a nitrogen flux, were then left under vacuum for one hour to obtain the two lipid films. The lipid films were then hydrated overnight with a solution of 5(6)-carboxyfluorescein (CF) in 30 mM HEPES buffer (pH 7.4) at room temperature and in the dark. The resulting suspensions contain multilamellar vesicles. To obtain small unilamellar vesicles (SUVs), each test tube was sonicated for 30 min over an ice bath twice. The resulting mixtures contained a large excess of non-encapsulated CF. To remove it, the suspension was purified via gel filtration (Sephadex G-7, Merck). The working solution (final concentration: 0.06 mM) of each SUV was obtained by diluting the corresponding mother solutions obtained with HEPES buffer (5 mM HEPES, 100 mM NaCl, pH 7.4). The final SUV solutions were stored in an ice bath and used within 24 h. To evaluate the peptide ability to modulate membrane permeability, CF leakage from the liposomes was measured via fluorescence spectroscopy on a Perkin Elmer (Waltham, MA, USA) MPF-66 spectrofluorimeter. The procedure was as follows: 2.5 mL of each SUV at 0.06 mM concentration was inserted in twelve disposable fluorescence cuvettes and fluorescence was recorded (λ_exc_ = 488 nm) for all of them at 520 nm (F_0_). Then, increasing volumes of each of the three peptides, dissolved in methanol, were added, and the fluorescence was recorded after 20 min of incubation time (t = 20 min) under the same experimental conditions (λ_exc_ = 488 nm; λ_obs_ = 520 nm) (F_t_). The total fluorescence intensity (F_T_) was then determined by adding 50 μL of an aqueous 10% *v/v* Triton X-100 solution to each cuvette. The formula %CF = (F_t_ − F_0_)/(F_T_ − F_0_) − 100 was used to obtain the percentage of CF released for all the [peptide]/[lipid] molar ratios (R^−1^) tested.

### 4.5. Antitumor Activity Studies

#### 4.5.1. Cell Lines

MDA-MB-231 (human triple-negative breast cancer), A549 (human lung adenocarcinoma), SK-OV-3 (human ovarian adenocarcinoma) and T24 (human transitional cell bladder carcinoma) cell lines were purchased from American Type Culture Collection (ATCC, Rockville, MD, USA). MUG-Mel2 (human melanoma) cell line was purchased from DSMZ (German Collection of Microorganisms and Cell Cultures, Braunschweig, Germany). MDA-MB-231 were grown in Dulbecco’s Modified Eagle Medium (DMEM) with Glutamax supplemented with 10% heat-inactivated fetal bovine serum (FBS) and antibiotics (100 U/mL streptomycin, 100 μg/mL penicillin G). A549 cells were cultured in F12K medium supplemented with 10% fetal bovine serum and antibiotics. SK-OV-3 cells were cultured in ATCC Modified RPMI-1640 medium supplemented with 10% fetal bovine serum and antibiotics. T24 cells were cultured in Eagle’s Minimum Essential Medium (EMEM) supplemented with non-essential amino acids, 1 mM sodium pyruvate, 10% fetal bovine serum and antibiotics. MUG-Mel-2 cells were cultured in RPMI-1640 medium supplemented with 20% fetal bovine serum and antibiotics.

All cell culture media and supplements were purchased from Life Technologies or Sigma-Aldrich (Munich, Germany), while sterile plasticware was purchased from Falcon^®^ (Corning, New York, NY, USA).

#### 4.5.2. In Vitro Cytotoxicity of Peptaibols

The cytotoxicity of To1-VX, To11-VX and VX peptides was assessed with the MTS assay (CellTiter 96^®^ AQueous One Solution Cell Proliferation Assay, Promega, Milan, Italy) in MDA-MB-231, A549, SK-OV-3, T24 and MUG-Mel-2 human cancer cells exposed to increasing concentrations (0–20 µM) of peptaibols for 24 h. Cells (8 × 10^3^ cells) were seeded in 96-well plates, and after 24 h, the medium was replaced with a fresh one containing peptaibols. For MTS assay, the medium was replaced with 100 μL of serum-free medium and 20 μL of the CellTiter 96^®^ reagent. After 60–90 min, the absorbance at 490 nm was measured with a Victor 3 (Perkin Elmer, Waltham, MA, USA) multimodal microplate reader and cell viability was expressed as a function of absorbance relative to that of control cells (considered as 100% viability). IC_50_ values and corresponding confidence intervals at 95% for each peptaibol were calculated from the relative dose–response curves using GraphPad Prism 9.5.

## Figures and Tables

**Table 1:** Primary structure of the pentadecaibin analogs that were synthesized and studied.

Acronym	Name	Sequence ^1^
VX	pentadecaibin	Ac-Aib-Gly-Ala-Leu-Aib-Gln-Aib-Val-Aib-Ala-Aib-Aib-Aib-Gln-Phol
To1-VX	[Toac^1^]- pentadecaibin	Ac-**Toac**-Gly-Ala-Leu-Aib-Gln-Aib-Val-Aib-Ala-Aib-Aib-Aib-Gln-Phol
To11-VX	[Toac^11^]- pentadecaibin	Ac-Aib-Gly-Ala-Leu-Aib-Gln-Aib-Val-Aib-Ala-**Toac**-Aib-Aib-Gln-Phol

^1^ Aib, α-aminoisobutyric acid; Toac, 2,2,6,6-tetramethylpiperidine-1-oxyl-4-amino-4-carboxylic acid; Phol, phenylalaninol; Ac, acetyl.

**Table 2:** Tips to perform a successful synthesis of Toac-containing pentadecaibin analogs.

Tip	
1	For Toac-containing pentadecaibins, the use of Gln residues without side-chain protection is recommended.
2	If acid-sensitive side-chain protections are used, cleave the fully protected peptide from the resin first, then remove protections via treatment with TFA 5% and TIS 2.5% (no water) for 7 h.
3	Choose the Toac position so that it never has to act as a nucleophile onto an incoming Aib.
4	Apply TFFH with at least 20’ preactivation for Aib/Toac onto Aib couplings. Reaction time: 24 h.
5	If TFA has to be used for peptide cleavage from resin, pour the solution from the reaction vessel on fresh CH_3_OH to avoid the esterification of the C-terminal 1,2-aminoalcohol

**Table 3:** IC_50_ (μM) values measured in MDA-MB-231, SK-OV-3, A549 and MUG-Mel2 cells exposed to peptaibols for 24 h.

IC_50_ (μM)			
Cancer Cell Line	VX [CI ^1^]	To1-VX	To11-VX
MDA-MB-231	8.79 [8.38–9.24]	7.98 [7.85–8.12]	8.04 [7.88–8.199]
SK-OV-3	11.09 [10.25–11.95]	9.56 [9.38–9.78]	9.64 [9.24–9.44]
A549	9.099 [8.50–9.97]	11.09 [8.38–14.30]	9.70 [9.005–10.86]
MUG-Mel2	14.24	10.05	13.6

^1^ CI, confidence interval at 95%.

## Data Availability

Data supporting reported results can be provided by the authors.

## Supplementary Materials

The supporting information can be downloaded at: https://www.mdpi.com/article/10.3390/ijms241713396/s1.
