# Supplementary material for: Synthesis, Conformational Analysis and Antitumor Activity of the Naturally Occurring Antimicrobial Medium-Length Peptaibol Pentadecaibin and Spin-Labeled Analogs Thereof
Source: Int J Mol Sci. 2023 Aug 29;24(17):13396. doi: 10.3390/ijms241713396 (PMC10487733; doi:10.3390/ijms241713396)
Supplement: Supplementary file 1 [file ijms-24-13396-s001.zip › ijms-2509256-supplementary.pdf]

Supporting Information

Synthesis, Conformational Analysis and Antitumor Activity of the Naturally Occurring Antimicrobial Medium-Length Peptaibol Pentadecaibin and Spin-Labeled Analogs Thereof

Laura Morbiato [1], Celeste Quaggia [1], Luca Menilli [2], Chiara Dalla Torre [1], Antonio Barbon [1] and Marta De Zotti [1,*]

[1] Department of Chemical Sciences, University of Padova, 35131 Padova, Italy; laura.morbiato.1@unipd.it (L.M.); celeste.quaggia@studenti.unipd.it (C.Q.); chiara.dallatorre.2@studenti.unipd.it (C.D.T.); antonio.barbon@unipd.it (A.B.)
[2] Department of Biology, University of Padova, 35131 Padova, Italy; luca.menilli@unipd.it
* Correspondence: marta.dezotti@unipd.it; Tel.: +39-049-827-5280

TABLE OF CONTENTS

1. HR-ESIMS spectra and a HPLC chromatogram of the crude peptides (figures S1-S5)
2. cw-EPR spectra (figure S6)
3. HPLC profiles and HR-ESIMS and ^{1}H NMR spectra for the new peptide sequences synthesized in the present work (figures S7-S12)
4. Biotage purification procedure for To1-VX (figure S13)

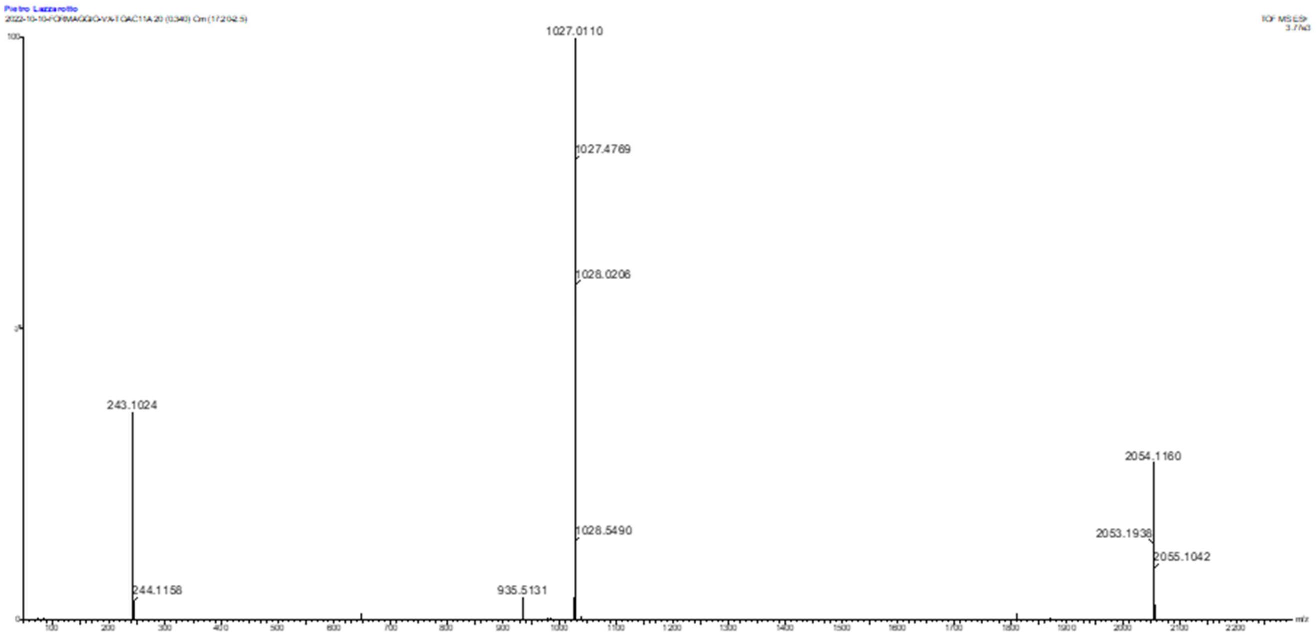

Figure S1. High Resolution Mass Spectrum (HRMS) acquired for [Gln(Trt)]To11-VX after cleavage from the resin with HFIP 30% in CH_2Cl_2 (DCM) for 1 hour. [MW_{calcd}:2052,14] [$(M+H]^{+}_{found}$=2053,1936 m/z [$(M+2H]^{2+}_{found}$= 1027,0110 m/z. MW TrtH = 243.1.

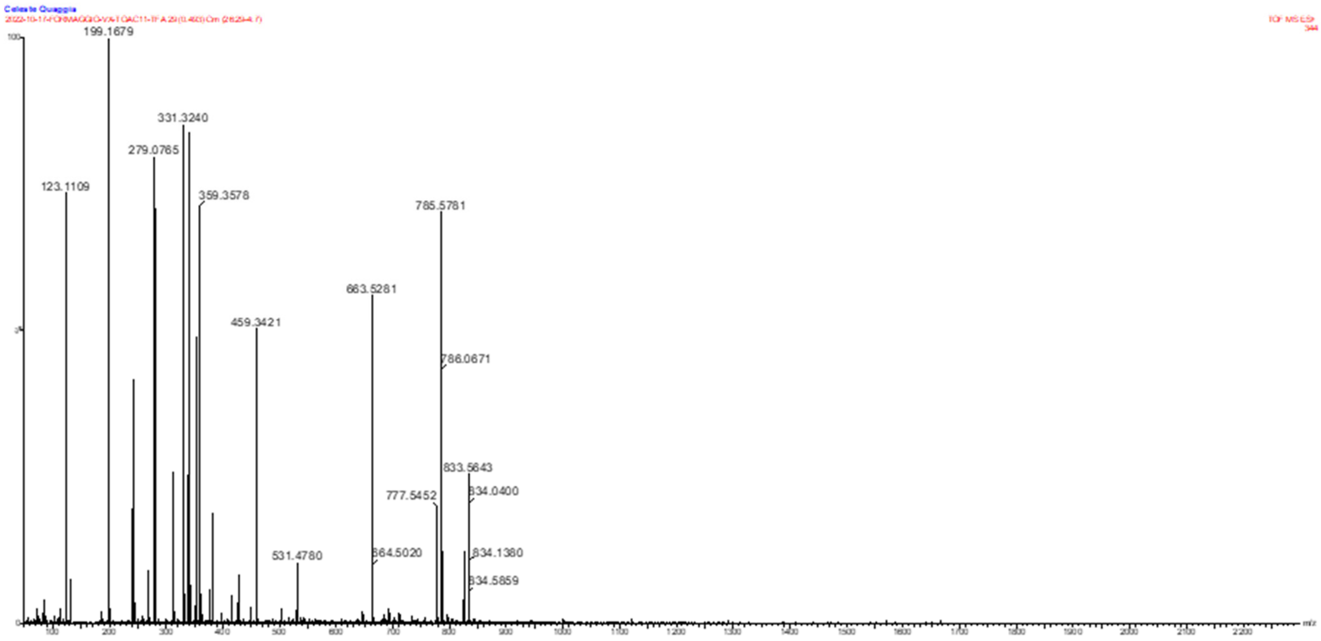

Figure S2: HRMS spectrum of the crude mixture of figure S1 after treatment with 20% TFA in DCM for 2 h 30 min. [MW_{calc}=1568.92]; $[M+2H]_{found}^{2+}$= 785,5781 m/z

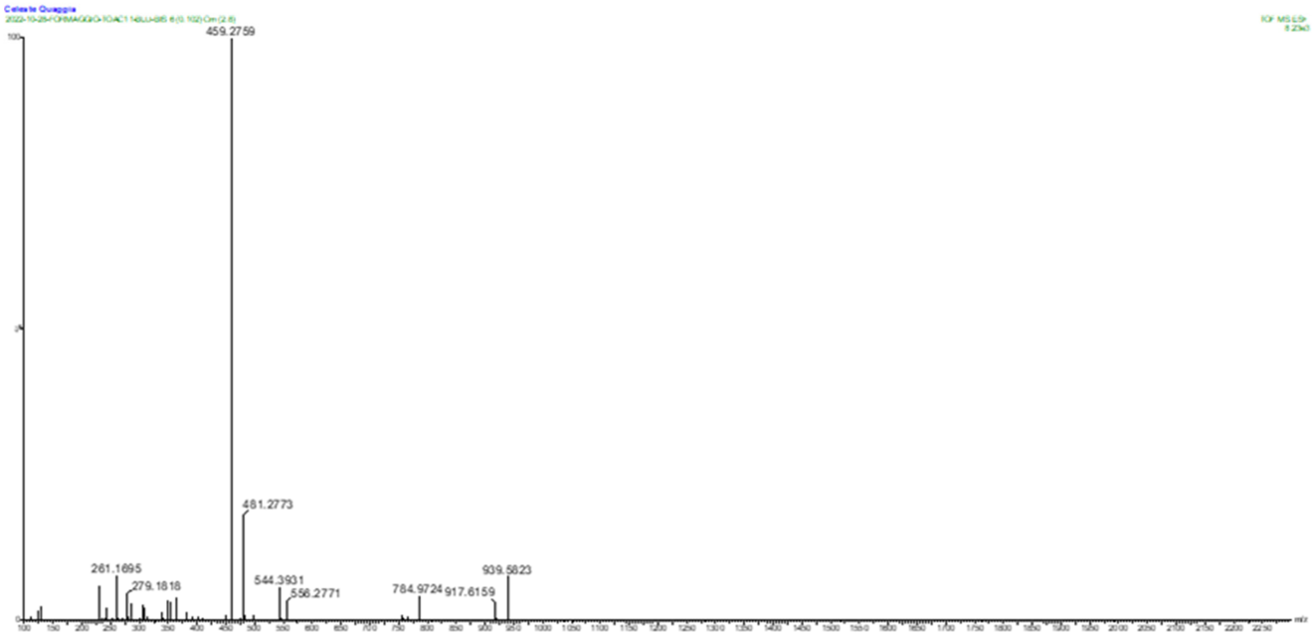

Figure S3: HRMS spectrum of the crude mixture of figure S1 after treatment with 10% TFA, 2,5%TIS and 2,5% H_2O for 16 hours.

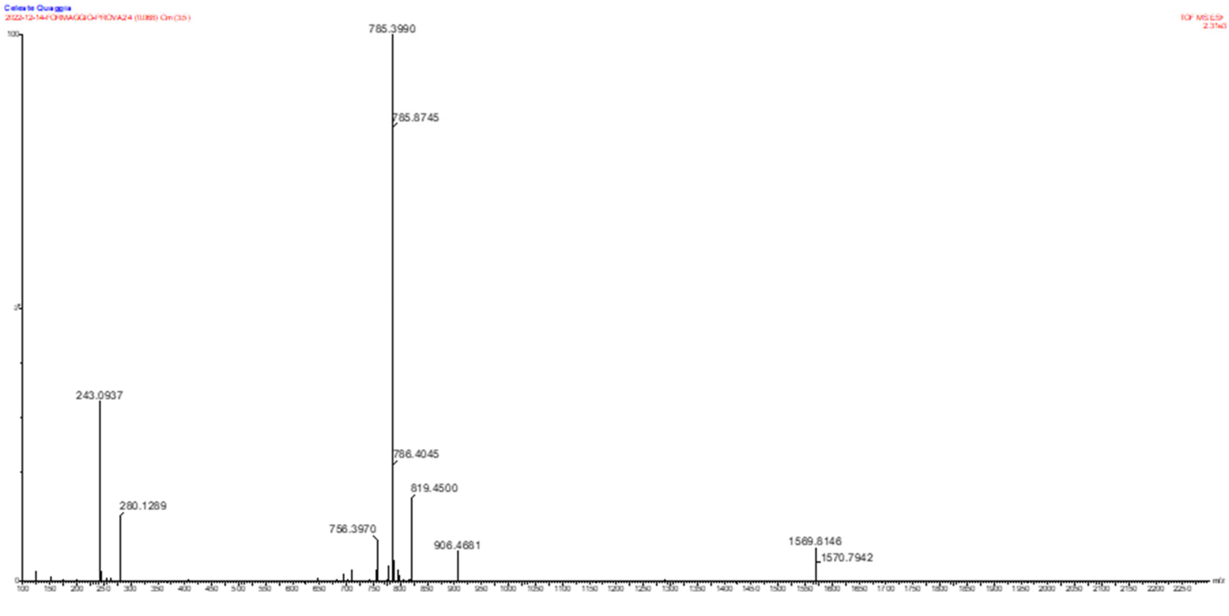

Figure S4: HRMS spectrum of the crude mixture of figure S1 after treatment with 5% TFA, 2,5%TIS, for 7 hours. [MW_{calcd}:1568,92]. $[M+H]^{+}_{found}$=1569,8146 m/z; $[M+2H]^{2+}_{found}$= 785,3990 m/z; $[M-58]^{2+}_{found}$= 756,3970 m/z.

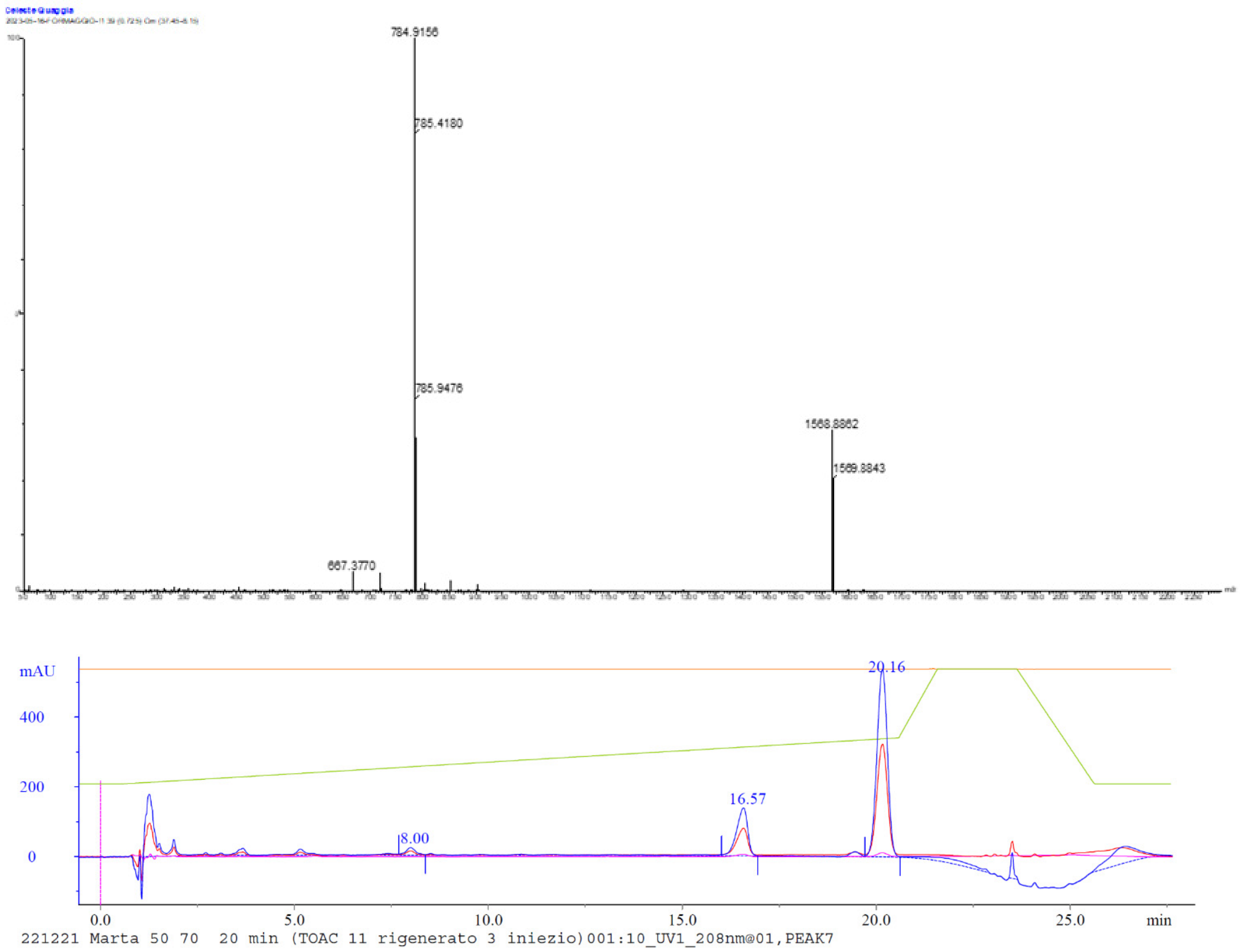

Figure S5. Representative HR-ESIMS spectrum and HPLC chromatogram (gradient: 50-70%B in 22min; R_t 20.16 min) of the crude product To11-VX. MW_{calcd} = [1567,92]; $[M+H]^{+}_{found}$=1568,8882 m/z $[M+ 2H]^{2+}_{found}$= 784,9156 m/z. The high crude purity was achieved by using Gln with unprotected side chains.

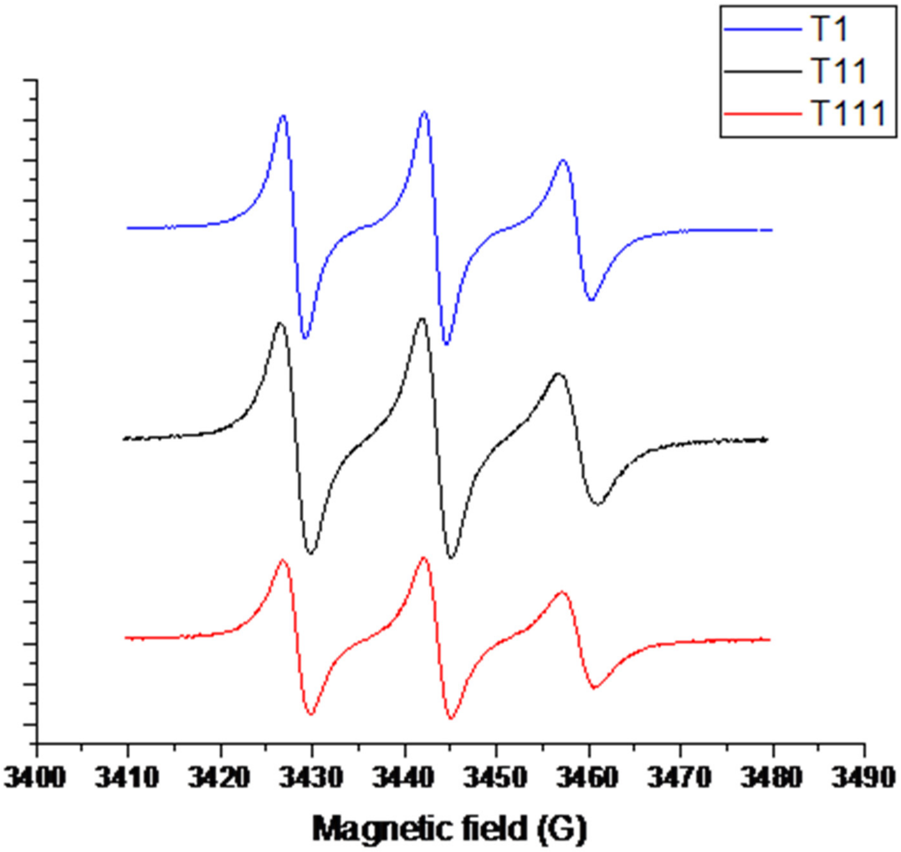

Figure S6. cw-EPR spectra of peptides: To1-VX (blue), To11-VX (black) and To1,11-VX (red), in CH_3OH. Peptide concentration: 1 x 10^{-4} M.

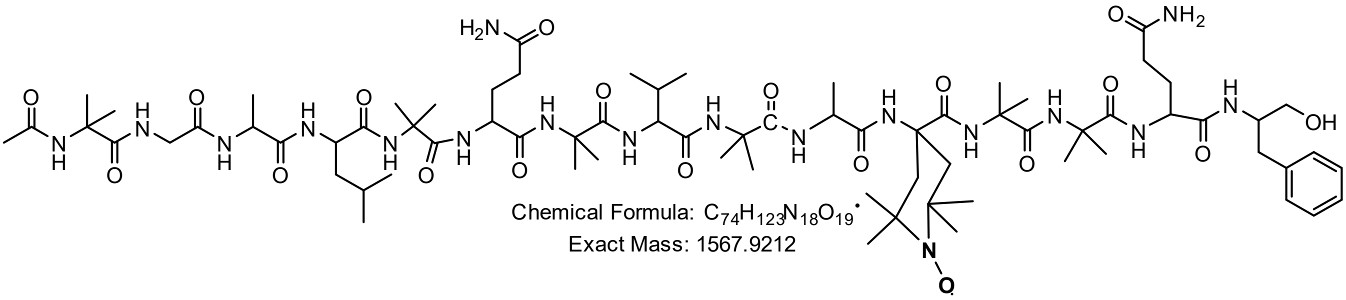

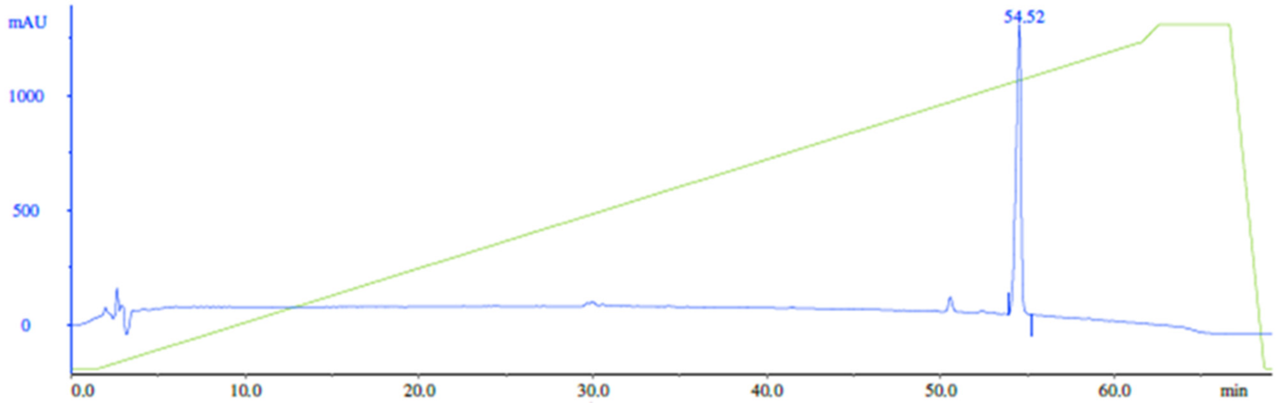

Figure S7. HPLC profile of purified To11-VX. Experimental conditions: Phenomenex C_{18} column (250x4.6mm 5μm). Gradient: 5-95 %B in 60 min. Purity: 94%

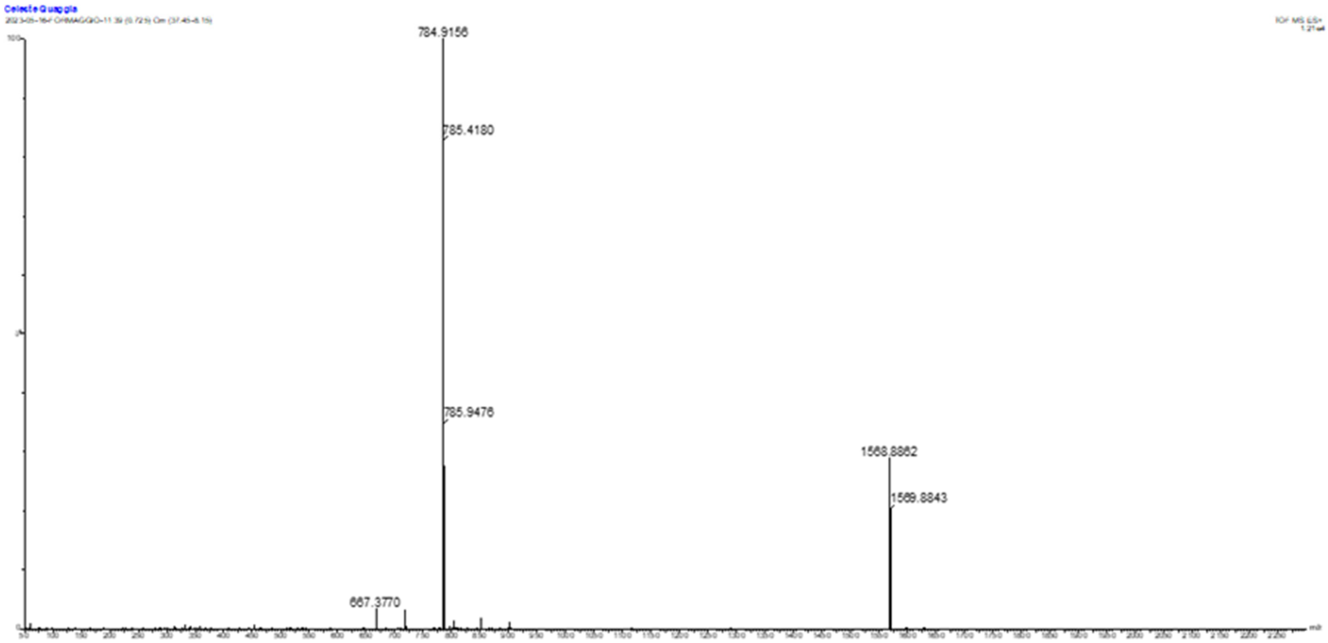

Figure S8. ESI HRMS spectrum of To11-VX. MW_{calc} 1567,9212; $[M+H]^{+}_{found}$=1568,8862; [M+ 2H]$^{2+}_{found}$= 784,9156.

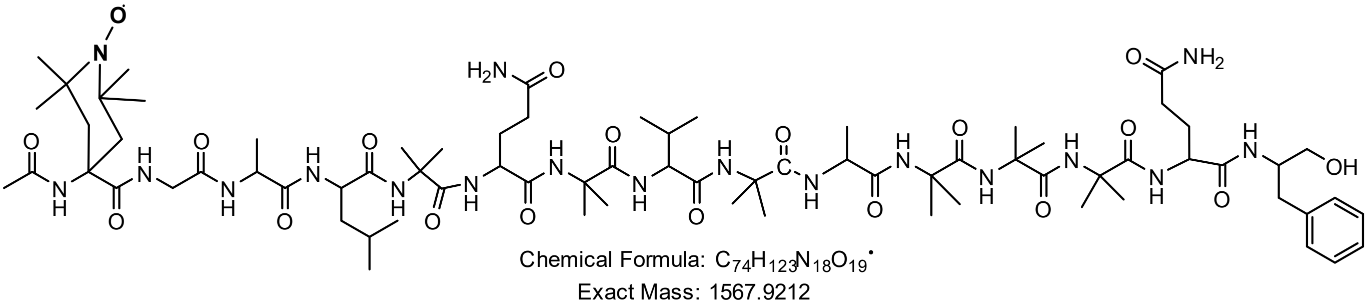

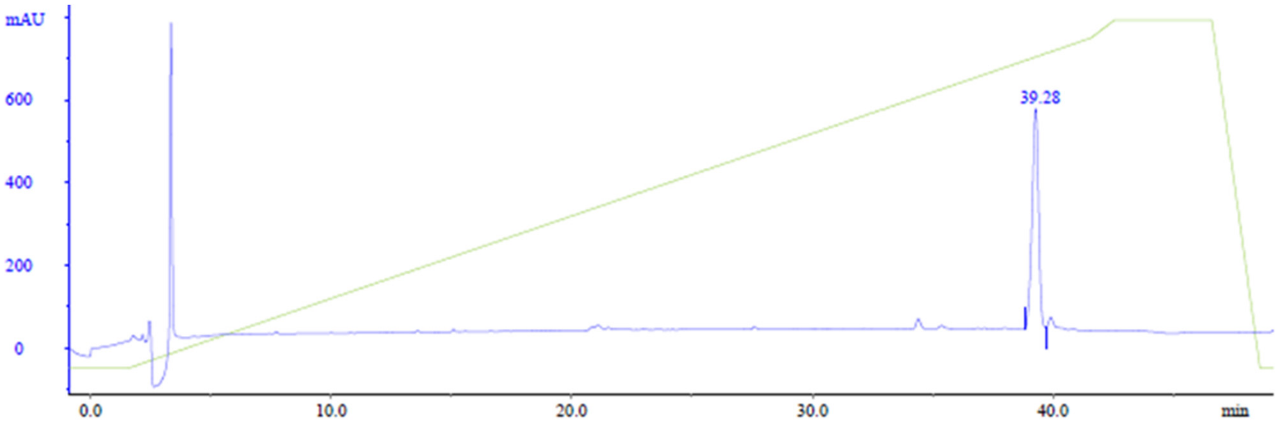

Figure S9. HPLC profile of purified To1-VX. Experimental conditions: Phenomenex C_{18} column (250x4.6mm 5µm); gradient: 5-95 %B in 40 min. Purity: 93%

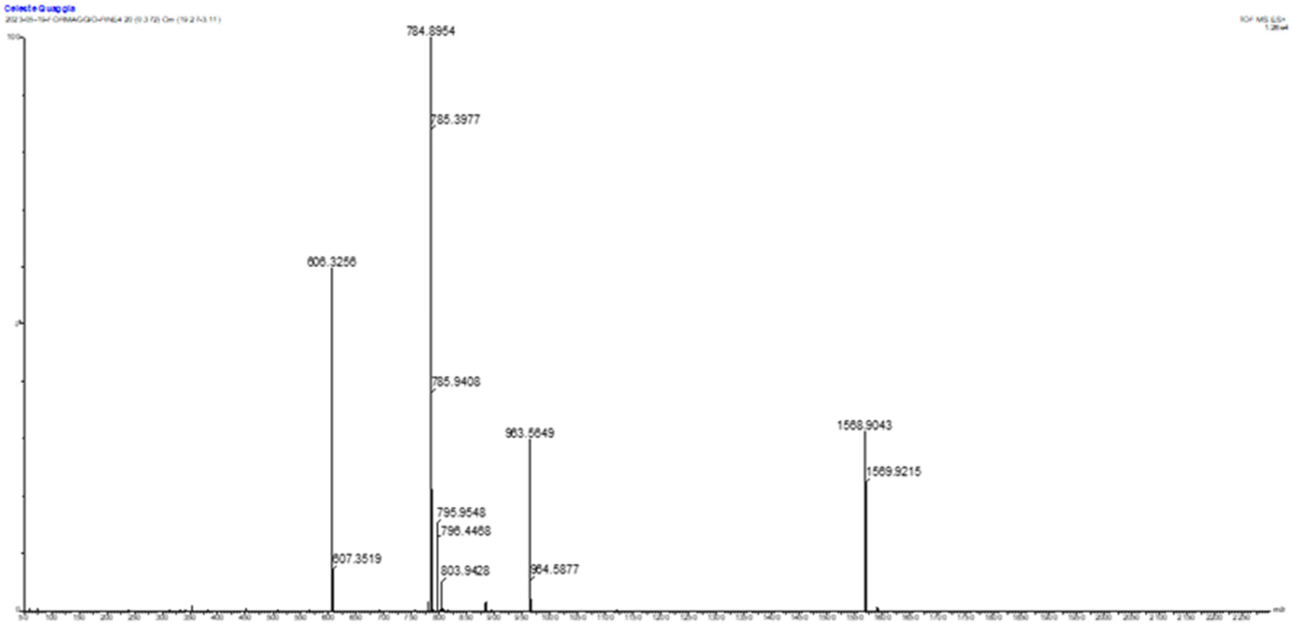

Figure S10. ESI HRMS spectrum of To1-VX. MW_{calc} 1567,9212; $[M+H]^{+}_{found}$=1568,9043; $[M+2H]^{2+}_{found}$= 784,8954; experimental mass fragments :605,32+962,56=1567,88.

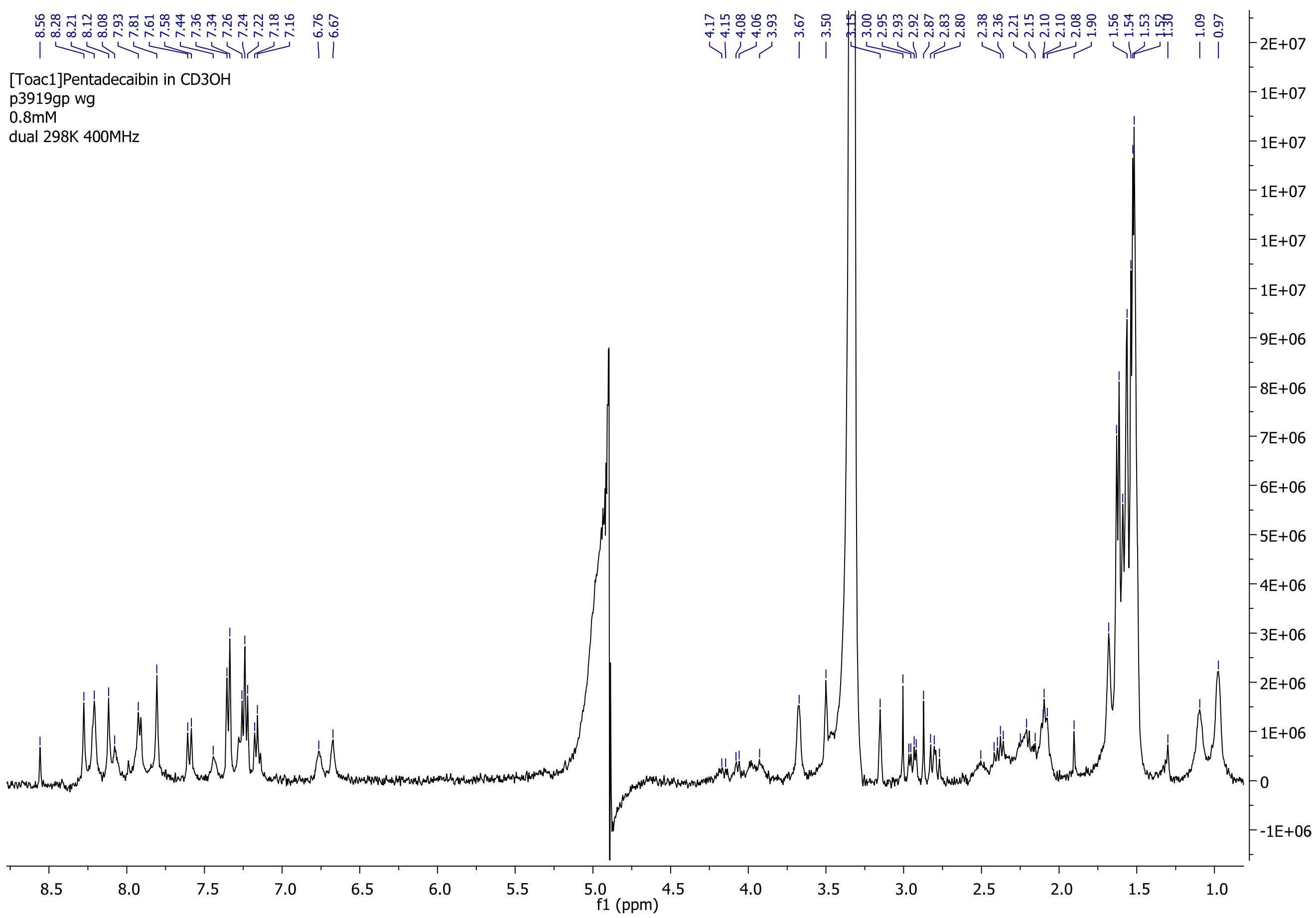

Figure S11. ^{1}H NMR spectrum of To1-VX in CD_3OH. Experimental conditions: 400MHz, 298K, peptide concentration: 0.8 mM.

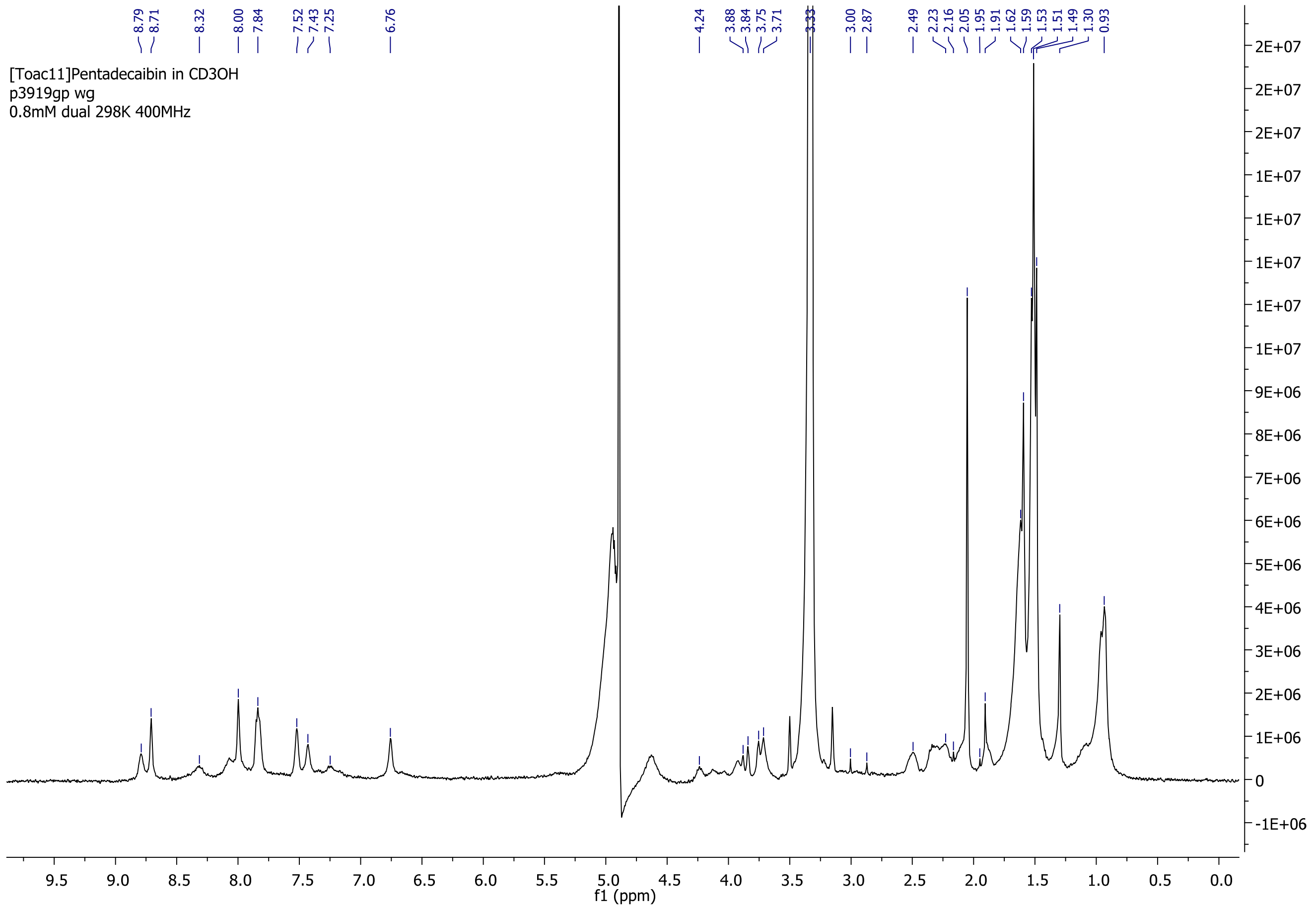

Figure S12. ^{1}H NMR spectrum of To11-VX in CD_3OH. Experimental conditions: 400MHz, 298K, peptide concentration: 0.8 mM.

Isolera Prime™ Fractions Report 2023-Jan-17 12.50

1.

**User**	MARTA	**Cartridge**	SNAP C18 12g
**Sample Name**	2023-Jan-17 12.50	**Rack Type**	18x150 mm
**Date**	2023-Jan-17 12.54	**Max Fraction Volume**	25 ml
**Method**	C18 VX TOAC1 rigenerato	**Solvent A**	Water
**Detection Mode**	UV1+UV2	**Solvent B**	Acetonitrile
**UV1 (Collection)**	206 nm		
**UV2 (Collection)**	260 nm		

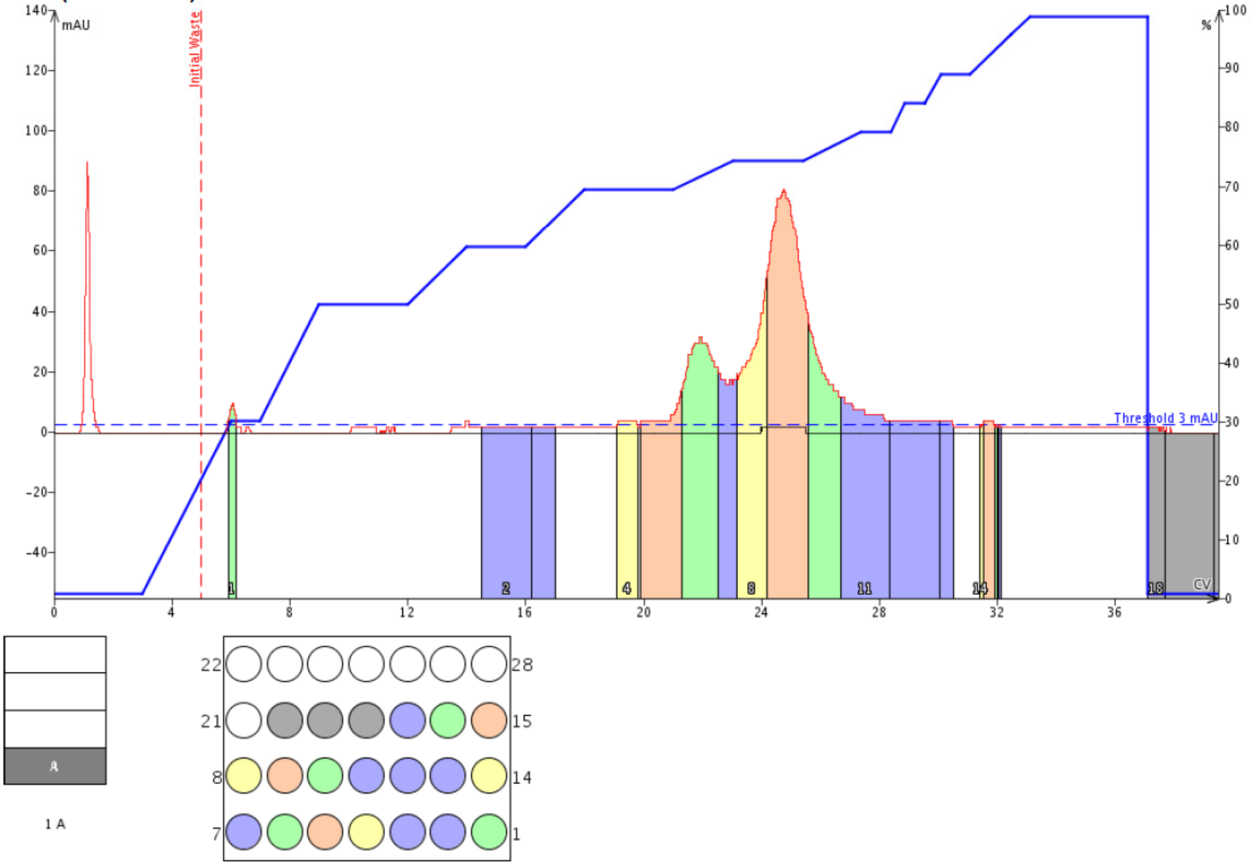

Figure S13. Report from the medium-pressure chromatographic purification performed on the crude sample of To1-VX. Biotage Isolera Prime instrument; flow rate, 12mL/min; C_{18} Duo Sfär column (12g).
